# Supplementary material for: Humic Substances Isolated from Recycled Biomass Trigger Jasmonic Acid Biosynthesis and Signalling
Source: Plants (Basel). 2023 Sep 1;12(17):3148. doi: 10.3390/plants12173148 (PMC10490330; doi:10.3390/plants12173148)
Supplement: Supplementary file 1 [file plants-12-03148-s001.zip › plants-2530646-Supplementary File Table 1.pdf]

Supplementary File Table S1: Primers used in this study.

Gene	Nomenclature	GenBank	Primer (5' to 3')	N° bases	Temperature°C
PDLa3	Phospholipase D alpha	Solyc08g066800.2	GTTACTGCCTTTGCACGTTT	20	57.51
			TGGAAATTGTACGTTCGACA	20	57.35
LOX2S	Lipoxygenase 2	Solyc01g006540	GCCACCAATAGATGATAAACCTC	23	57.05
			CAATGAGTTCTTAGCCAGTGAC	22	57.06
OPR3	12-oxiphytodienoate reductase 3	Solyc07g007870.2	CTCCACAGTCCACCATTCAT	20	57.20
			GGAGAGAAAAGGGGATTGTT	20	58.69
AOS2	Allene oxide synthase 2	Solyc11g069800.1	CGAGTTGATCCTCCGGTAGC	20	59.97
			CCCGGCCGGTCAAAAATTTT	20	59.98
MYC2	Transcription factor	Solyc08g076930.1	CAGTTTTGCCTTCTTCGGGC	20	60.04
			TTCGCTGGCTTTCTACCTCG	20	60.11
ARF6a	Auxin response factors	Solyc00g196060.2	TGACGACAGGATGGAGTGTG	20	59.40
			GGCACGTCGTATCCCCAAA	19	60.08
ARF8a	Auxin response factors	Solyc02g037530.2	CTGCTCAAACCCAAATGCTGTC	22	60.61
			GGTAAGTGTGTTGGTGAGCCTG	22	61.39
JAR1	Jasmonoyl-isoleucine synthetase	Solyc10g011660.2.1	AGGAAAATCTCGTAGGCGTG	20	57.70
			TCTGTAACGATATAAACCTGCG	22	58.10
JAZ	Jasmonate ZIM Domain-containing protein	Solyc11g011030.2	CTAAAGGAGCACTTGCTATGG	21	56.49
			CCAATGAACGCTTGACGA	18	57.34

ETR	Etileno response sensor (ETR)	Solyc06g053710	CTGGTTCAGTTGATGCAAGG	20	57.00
			ACACCTTGGAGGAGTGAG	18	56.97
CEVI57	Proteinase inhibitor II	Solyc06g053711	TCCTAACATTTGCCCCTCATA	21	58.39
			TCCTTCACACACAAACTCTCC	21	57.81
ACT4	Actin (endogenous control)	Solyc06g053712	GGTCCCTCTATTGTCCACAG	20	57.01
			TGCATCTCTGGTCCAGTAGGA	21	59.99
